# Beetroot, watermelon and ginger juice supplementation may increase the clinical outcomes of Intracytoplasmic Sperm Injection cycles

**DOI:** 10.5935/1518-0557.20230012

**Published:** 2023

**Authors:** Gabriela Halpern, Daniela Paes de Almeida Ferreira Braga, Christina Morishima, Amanda Souza Setti, Assumpto Iaconelli Setti Jr., Edson Borges Jr.

**Affiliations:** 1 Fertility Medical Group, Av. Brigadeiro Luiz Antônio, 4545. São Paulo – SP, Brazil. Zip: 01401-002; 2 Instituto Sapientiae – Centro de Estudos e Pesquisa em Reprodução Assistida Rua Vieira Maciel, 62. São Paulo – SP, Brazil. Zip: 04503-040

**Keywords:** beetroot, watermelon, ginger, superfoods, pregnancy

## Abstract

**Objective:**

To prove the hypothesis that beetroot, watermelon and ginger juice supplementation improves the endometrial receptivity and clinical outcomes of intracytoplasmic sperm injection (ICSI) cycles.

**Methods:**

This prospective randomized study enrolled 436 female patients undergoing ICSI cycles from January/2018 to June/2021, in a private university–affiliated IVF center. Female patients were randomized in a 1:3 ratio to either Control (n=109) or Supplementation Group (n=327). All patients received nutritional orientation before the beginning of the treatment. Participants in the Supplementation Group were instructed to intake a daily dose of homemade juice, prepared with fresh beetroot, watermelon and ginger, from the day of embryo transfer until the day of pregnancy test, while patients in Control Group did not follow the juice protocol. Generalized Linear Models, adjusted for potential confounders (female age, body mass index - BMI, endometrial thickness upon embryo transfer, and number of transferred embryos), followed by Bonferroni post hoc test for the comparison of means between groups, were used to investigate the impact of juice supplementation on the clinical outcomes of ICSI.

**Results:**

Patients and cycles characteristics were equally distributed among Supplementation and Control groups. Implantation rate (25.2% vs. 20.5%, p<0.001) and clinical pregnancy rate (41.0% vs. 22.0%, p=0.039) were significantly higher in the Supplementation compared to the Control group.

**Conclusions:**

The use of beetroot, watermelon and ginger juice may be considered a promising strategy for improving clinical outcomes in assisted reproductive technology (ART), without any side effects.

## INTRODUCTION

It is estimated that about 50 to 80 million people around the world experience infertility during their reproductive life (Dyer *et al.,* 2016). Since the first reported pregnancy following *in vitro* fertilization (IVF) (Steptoe & Edwards, 1978), the use of assisted reproductive technology (ART) has increased dramatically worldwide and has made pregnancy possible for many infertile couples (Sullivan *et al.,* 2013). However, its efficacy in terms of take home baby is still low (Sunkara *et al.,* 2016).

Many factors may influence the implantation of in vitro-produced embryos. Recently special attention has been given to patient’s lifestyle. There is a growing body of literature demonstrating that weight (van Oers *et al.,* 2016), diet (Braga *et al.,* 2012; 2015; Best *et al.,* 2017; Karay-iannis et al., 2018; Setti *et al.,* 2018), exercise (Ferreira *et al.,* 2010; Palomba *et al.,* 2014), smoking (Borges *et al.,* 2018; Budani *et al.,* 2018; Mínguez-Alarcón *et al.,* 2018), alcohol consumption (Borges *et al.,* 2018; Mínguez-Alarcón *et al.,* 2018), and others significantly decreases the chance of clinical pregnancy.

Different foods have biological activities that may harm or benefit health. In recent years there has been a growing interest in the biological activity of red beetroot (*Beta vulgaris rubra*) as a source of antioxidants and micronutrients including potassium, betaine, sodium, magnesium, vitamin C and perhaps most notably nitrate (Clements *et al.,* 2014), leading to increased nitric oxide (NO) availability (Lundberg *et al.,* 2008). Therefore, its intake has emerged as a strategy to prevent and manage pathologies associated with diminished NO bioavailability. Beetroot is also being considered as a promising therapeutic treatment in pathologies associated with oxidative stress and inflammation (Clifford *et al.,* 2015). Previous data demonstrate that a single dose of 140 mL of beetroot juice consumption improves vascular endothelial function (Volino-Souza *et al.,* 2018).

It has also been suggested that increased l-arginine levels in the circulation may represent a potential therapeutic mechanism to increase NO synthesis and bioavailability. However, oral l-arginine supplementation is largely ineffective due to gastrointestinal and hepatic extraction of l-arginine (Allerton *et al.,* 2018). Alternatively, oral l-citrulline supplementation consistently increases plasma and tissue levels of l-arginine and NO bioavailability (Moinard *et al.,* 2008; Schwedhelm *et al.,* 2008). I-Citrulline is a neutral, non-essential alpha-amino acid, rarely found in food, but highly concentrated in watermelon (*Citrullus lanatus*) (Curis *et al.,* 2005).

In addition to the beetroot and watermelon, compounds found in ginger (*Zingiber officinale*), particularly the 6-Gingerol, has been reported to possess a variety of biological properties including anti-oxidant, anti-inflammatory, anti-platelet aggregation, antifungal, antimicrobial, and anticancer activities. Ginger is also known to decrease the activities of angiotensin-1 converting enzyme and arginase and increased the level of NO (Mao *et al.,* 2019).

Embryo implantation depends on the presence of a viable embryo in a receptive endometrium. The endometrium is a highly dynamic tissue that undergoes cyclic cellular proliferation, differentiation, and immune cell trafficking in response to changing circulating ovarian-derived steroids (Valdes *et al.,* 2017). Endometrium angiogenesis play a crucial role during the menstrual cycle, particularly around the time of embryo implantation (Gargett & Rogers, 2001). Any alteration in endometrial vascularization may lead to unsuccessful implantation.

The goal for the present study was to evaluate weather beetroot, watermelon and ginger juice supplementation would improve the endometrial receptivity and clinical outcomes of intracytoplasmic sperm injection (ICSI) cycles.

## MATERIALS AND METHODS

### Study design

This prospective randomized study enrolled 436 female patients undergoing ICSI cycles from January/2018 to June/2021, in a private university-affiliated IVF center. Female patients were randomized in a 1:3 ratio to either Control (n=109) or Supplementation Group (n=327). All patients received nutritional orientation before the beginning of the treatment. Participants in the Supplementation Group were instructed to intake a daily dose of homemade juice, prepared with fresh beetroot, watermelon and ginger, from the day of embryo transfer until the day of pregnancy test, while patients in Control Group did not follow the juice protocol. The impact of juice supplementation on the clinical outcomes of ICSI was investigated.

Clinical pregnancy was defined as the presence of a gestational sac that could be visualized using ultrasound 4-6 weeks after embryo transfer, and miscarriage was defined as pregnancy with a total loss of gestational sacs before 20 weeks’ gestation.

Written informed consent was obtained, in which patients agreed to share the outcomes of their own cycles for research purposes, and the study was approved by the local institutional review board.

### Controlled ovarian stimulation and laboratory procedures

Controlled ovarian stimulation was performed using recombinant follicle-stimulating hormone (r-FSH, Gonal-F®; Serono, Geneva, Switzerland), with pituitary blockage using a gonadotropin-releasing hormone (GnRH) antagonist, cetrorelix acetate (Cetrotide®; Merck KGaA, Serono, Geneva, Switzerland).

Follicular growth was monitored using transvaginal ultrasound examination starting on day 4 of gonadotropin administration. When adequate follicular growth and serum estradiol levels were observed, leuprolide acetate (Lupron®; TAP Pharmaceuticals, North Chicago, IL, United States) was administered to trigger the final follicular maturation. The oocytes were collected 35 hours later through transvaginal ultrasound ovum pickup.

The recovered oocytes were assessed to determine their nuclear status, and those in metaphase II were submitted to intracytoplasmic sperm injection (ICSI) following routine procedures (Palermo *et al.,* 1997).

After ICSI oocyte/embryos were cultured until day five when one or two blastocysts were transfer.

### Embryo morphology evaluation

Embryo morphology was assessed: (i) 16 to 18 hours post-ICSI ± 1hour, (ii) 44 hours ± 1hour post-ICSI, for day two evaluation (iii) 68 hours ± 1hour post-ICSI, for day three evaluation (iv) 116 hours ± 2hour post-ICSI for day five evaluation and (v) 120 hour ± 2hour post-ICSI for late day five evaluation (transfer time). Embryo morphology was performed using an inverted Nikon Diaphot microscope (Eclipse TE 300; Nikon, Tokyo, Japan) with a Hoffmann modulation contrast system under 400X magnification and was considere to choose best embryos for transfer.

### Statistical analysis

The sample size calculation suggested that 265 cycles at least would be enough to demonstrate a 20% effect with 90% power and 5% significance level considering as primary outcome clinical pregnancy rate. An increase of 33% in sample size was required due to a 3:1 randomization. Sample size calculation was performed using G*Power 3.1.7.

Generalized Linear Models, adjusted for potential confounders (female age, FSH dose used for controlled ovarian stimulation, endometrial thickness upon embryo transfer, number of transferred embryos and high quality embryos rate), followed by Bonferroni post hoc test for the comparison of means between groups were used to investigate the impact of juice supplementation on the clinical outcomes of ICSI.

Data are expressed as mean ± standard error for continuous variables or percentages for dichotomous variables, and *p*-values. *P*-value was significant at 5% level (<0.05). The analysis was performed using SPSS Statistics 21 (IBM, New York, New York, USA).

## RESULTS

### Patient and cycle characteristics

Similar maternal and paternal ages, maternal BMI, estradiol level on hCG trigger day, FSH dose used for controlled ovarian stimulation, number of follicles, number of retrieved and mature oocytes, mature oocytes rate, fertilization rate, high quality embryo rate, blastocyst development rate, number of transferred embryos and endometrial thickness were observed between the groups (Table 1).

**Table 1:** Patient and cycle characteristics for the positive and negative pregnancy groups.

	Supplementation Group (n=222)	Control Group (n=74)	p value
Maternal age (years)	36.8±5.3	36,2±5,7	0.152
Paternal age (years)	39.2±9.1	38,9±5.3	0.284
BMI (Kg/m^2^)	24.8±0.6	24.6±0.5	0.695
FSH dose (mIU/ml)	2654.3±95.3	2451.0±50.2	0.521
Estradiol (pg/ml)	2475.6±251.6	2541.0±451.4	0.541
Aspirated follicles (n)	15.9±2.5	17.8±1.4	0.500
Retrieved oocytes (n)	12.9±0.6	12.1±0.8	0.435
Mature oocytes (n)	8.6±0.6	9.5±1.0	0.506
Mature oocytes rate (n)	74.9±1.6	74.7±1.8	0.839
Fertilization rate (%)	82.5±1.2	85.3±2.5	0.845
High-quality embryo rate (%)	45.5±2.0	46.9±3.5	0.541
Blastocyst formation rate	48.7±2.1	52.4±5.5	0.214
Transferred embryos	2.0±0.0	1.8±0.1	0.142
Endometrial thickness (mm)	10.2±0.2	10.2±0.3	0.838

Values described as mean or percentage (%) ± standard error. BMI: Body mass index.

### Influence of juicy supplementation of ICSI outcomes

Implantation rate and clinical pregnancy rate were significantly higher in the Supplementation compared to the Control group, while the miscarriage rate didn’t differ among the groups (Table 2).

**Table 2:** Generalized linear model results for the comparison of implantation, pregnancy and miscarriage rate between Supplementation and Control groups.

	Supplementation Group (n=222)	Control Group (n=74)	p value
Implantation rate (%)	25.2±0.36	20.5±0.60	<0.001
Pregnancy rate (%)	41.0%±7.1	22.0%±7.8	0.039
Miscarriage rate (%)	17.0%±5.0	25.0%±11.7	0.489

Values described as mean or percentage (%) ± standard error.

## DISCUSSION

Infertility has considerably increased over the past decades (Bushnik *et al.,* 2012; Luke, 2017). Besides the trend among women in developed countries to delay motherhood (Datta *et al.,* 2016), lifestyles factors may be pointed as reasons for the impaired reproduction potential (Harley *et al.,* 2008; Boots & Stephenson, 2011; Braga *et al.,* 2012; 2015; Practice Committee of the American Society for Reproductive Medicine, 2012; Buck Louis, 2014). For the present study weather beetroot, watermelon and ginger juice supplementation would improve clinical outcomes of ICSI cycles, was evaluated. The results showed that both implantation and clinical pregnancy rate were significantly higher when the juice was taken compared to control group. Additionally a significant difference in miscarriage rate was noted between the groups.

Subjects in the supplementation group were oriented to take a daily dose of the juice, from the day of embryo transfer until the day of pregnancy test, a critical period determining implantation of embryo in uterus. Molecular and genetic evidence indicates that ovarian hormones together with locally produced signaling molecules, function through autocrine, paracrine and juxtracrine interactions to specify the complex process of implantation (Zhang *et al.,* 2013). Growth factors, particularly the endothelial growth factor (VEGF), are involved in a fundamental process in embryo implantation, the angiogenesis (Chen *et al.,* 2017).

It has been speculated that recurrent reproductive failure, including recurrent miscarriage (Chen *et al.,* 2016) and recurrent implantation failure (Goodman *et al.,* 2008) after IVF are two events where abnormal endometrial angiogenesis may occur.

The cellular processes of angiogenesis involves hypoxia which induces the production of NO and the expression of VEGF and angiopoietin-1 and -2, which interact with extracellular matrix proteases to increase the permeability of the capillary vessel wall. Increased permeability leads to the escape of plasma proteins and fibrinogen from capillary vessels, promoting the formation of transient extracellular matrix, which supports the growth of new vessels (de Castro Junior *et al.,* 2006; Lin *et al.,* 2016).

The role of NO for the vascular health has been extensively studied. It acts as a hormone and its activities are generally beneficial. For example, it is a powerful generator of vasodilation by suppressing vascular smooth muscle contraction. Nitric oxide also inhibits platelet aggregation and the adhesion of leukocytes to endothelia (Litwack, 2014). Consequently, NO becomes a substance of interest in therapeutic applications.

The most recognized pathway for NO generation is the oxidation of L-arginine catalyzed by the NO synthase enzymes to yield NO and L-citrulline (Moncada & Higgs, 1991). Although traditionally recognized as a product of the NO synthase-mediated oxidation of L-arginine, recent evidence suggests that L-citrulline can be recycled back to L-arginine (Haines *et al.,* 2011) and is more effective than orally ingested L-arginine (Osowska *et al.,* 2004; Schwedhelm *et al.,* 2008). It has also been reported that L-citrulline supplementation can increase the activity of NO synthase (Wijnands *et al.,* 2012) and increase NO bio-markers (Schwedhelm *et al.,* 2008). Therefore, short-term supplementation with L-citrulline might represent an effective dietary intervention to increase NO synthase.

Watermelon juice is a particularly rich source L-citrulline. It has been reported that watermelon juice contains ~2.33g of L-citrulline per L (Tarazona-Díaz *et al.,* 2013). Moreover, watermelon contains lycopene, a lipophilic, unsaturated carotenoid, also known to improve vascular health (Gajendragadkar *et al.,* 2014) by several different mechanisms (Mozos *et al.,* 2018).

The watermelon is not the only food recognized to improve vascular heath, in recent years the beetroot has attracted much attention as a health promoting functional food. Interest in beetroot has been primarily driven by the discovery that sources of dietary nitrate may have important implications for managing cardiovascular health (Lundberg *et al.,* 2008). Beetroot is also rich in several other bioactive compounds that may provide health benefits, particularly for disorders characterized by chronic inflammation (Clifford *et al.,* 2015).

In fact, in addition to the effect on vascular health, the intake of both beetroot and watermelon has important antioxidant effects. In line with this, another superfood, the ginger has been reported to possess multiple biological activities, including antioxidant and anti-inflammatory (Mao *et al.,* 2019).

The use of this functional food has been widely studied as a source of energy during exercise (Barlow *et al.,* 2018; Ridwan *et al.,* 2019) or for the prevention and management of chronic diseases (Zhang *et al.,* 2016), but not for reproductive health. Considering that implantation failure may be a consequence of a diminished endometrial receptivity due to, among other causes, a weak angiogenesis or excessive oxidative stress, we have hypothesized that the intake of foods such as beetroot, watermelon and ginger would improve endometrial vascularization, reduce oxidative stress and therefore improve embryo implantation in women undergoing ART.

The intake of beetroot, watermelon and ginger juice significantly improved implantation and pregnancy rates. It could be argued that among so many variables influencing embryo implantation, the intake of some specific foods for such a short period of time could not be the reason for the improved clinical outcomes. However, NO action is rapid as it remains in the blood for only seconds and its short term effect in human have been already demonstrated (Bailey *et al.*, 2016; Porcelli *et al.,* 2016).

Moreover, previous evidence has suggested that there is an alteration of endometrial receptivity in patients with recurrent implantation failure. Women with repeated donor insemination failures have altered endometrial progression in relation to their menstrual cycle (Li *et al.,* 1993). Hysteroscopy studies have shown that 18%-50% of recurrent implantation failure patients have abnormalities of the uterine cavity (Margalioth *et al.,* 2006), while temporal displacement of the window of implantation has been described in one out of four repeated implantation failure patients (Ruiz-Alonso *et al.,* 2013). Therefore our evidence suggests that the intake of beetroot, watermelon and ginger juice from the embryo transfer day would have improved the endometrial angiogenesis and microenvironment by and increased NO, lycopene and antioxidants bioavailability favoring embryo implantation.

## CONCLUSIONS

Recently, lifestyle behaviors such as dietary intake, has been pointed as the strongest influencing factors related to human health. Researchers have put a lot of effort into understanding the impact of the selective use of isolated nutrients and nutritional supplements to enhance human health and functional capacity. Our results demonstrated that the use of specific nutrients may also improve reproductive health. The use of beetroot, watermelon and ginger juice may be considered a promising strategy for improving clinical outcomes in ART, without any side effects.
